# The immunoglobulin tail tyrosine motif upgrades memory-type BCRs by incorporating a Grb2-Btk signalling module

**DOI:** 10.1038/ncomms6456

**Published:** 2014-11-21

**Authors:** Niklas Engels, Lars M. König, Wiebke Schulze, Daniel Radtke, Kanika Vanshylla, Johannes Lutz, Thomas H. Winkler, Lars Nitschke, Jürgen Wienands

**Affiliations:** 1 Institute of Cellular and Molecular Immunology, Georg-August-University of Göttingen, Medical Faculty, Humboldtallee 34, 37073 Göttingen, Germany; 2 Chair of Genetics, Department of Biology, Friedrich-Alexander-University Erlangen-Nürnberg, Staudtstrasse 5, 91058 Erlangen, Germany; 3 Hematopoiesis Unit, Department of Biology, Nikolaus-Fiebiger-Center for Molecular Medicine, Friedrich-Alexander-University Erlangen-Nürnberg, Glückstrasse 6, 91054 Erlangen, Germany

## Abstract

The vigorous response of IgG-switched memory B cells to recurring pathogens involves enhanced signalling from their B-cell antigen receptors (BCRs). However, the molecular signal amplification mechanisms of memory-type BCRs remained unclear. Here, we identify the immunoglobulin tail tyrosine (ITT) motif in the cytoplasmic segments of membrane-bound IgGs (mIgGs) as the principle signal amplification device of memory-type BCRs in higher vertebrates and decipher its signalling microanatomy. We show that different families of protein tyrosine kinases act upstream and downstream of the ITT. Spleen tyrosine kinase (Syk) activity is required for ITT phosphorylation followed by recruitment of the adaptor protein Grb2 into the mIgG-BCR signalosome. Grb2 in turn recruits Bruton’s tyrosine kinase (Btk) to amplify BCR-induced Ca^2+^ mobilization. This molecular interplay of kinases and adaptors increases the antigen sensitivity of memory-type BCRs, which provides a cell-intrinsic trigger mechanism for the rapid reactivation of IgG-switched memory B cells on antigen recall.

## Abstract (web summary)

How class-switched memory B cells signal to respond robustly to recurring pathogens is incompletely understood. Here the authors show that immunoglobulin tail tyrosine motifs in membrane-bound immunoglobulin isotypes of class-switched cells amplify signalling in memory B cells, in a mechanism that includes distinct kinases and adaptor proteins.

## Graphical abstract

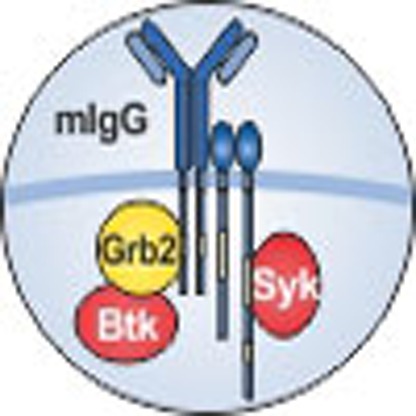

Humoral immunity is based on the establishment of protective antibody titres as well as the formation of long-lived memory B cells that are poised to swiftly differentiate into antibody-producing plasma cells on antigen reencounter^1^,^2^. Memory B cells express either membrane-bound IgM (mIgM) or class-switched mIg isotypes, most notably mIgGs, as part of the B-cell antigen receptor (BCR) on their cell surface^3^,^4^. However, mIgG-expressing memory B cells are preferentially reactivated over mIgM-expressing cells on antigen recall^4^,^5^. Recent evidence indicated that BCR-intrinsic signal amplification of mIgG-containing BCRs is involved in this process^6^,^7^.

The minimal BCR unit consists of an mIg molecule that non-covalently associates at a 1:1 stoichiometry with a heterodimer of mIg-associated α and β transmembrane proteins (Igα/β, CD79a/b)^8^,^9^. Igα and Igβ each contain one copy of the immunoreceptor tyrosine-based activation motif (ITAM) in their cytoplasmic domains that serves to recruit and activate cytosolic protein tyrosine kinases (PTKs) of the Src and Syk/ZAP70 families^10^,^11^. In addition, Igα contains an evolutionarily conserved non-ITAM tyrosine residue, Y204, that on phosphorylation recruits the central B-cell adaptor protein SLP65 (BLNK) via its Src homology (SH) 2 domain^12^,^13^,^14^. Phosphorylation of SLP65 by activated Syk allows the formation of a multimolecular protein complex consisting of the key enzymes for BCR-induced Ca^2+^ mobilization, the PTK Bruton’s tyrosine kinase (Btk) and phospholipase C-γ2 (PLC-γ2), both of which bind to phospho-SLP65 via their SH2 domains^15^,^16^. The formation of this complex is critical for activation of PLC-γ2 by Btk and subsequent generation of the second messengers diacylglycerol and inositol-1,4,5-trisphosphate (IP3) by cleavage of the membrane lipid phosphatidylinositol-4,5-bisphosphate. IP3 directly activates Ca^2+^ channels in the endoplasmic reticulum (ER) and plasma membranes and thus controls the intensity and duration of BCR-induced Ca^2+^ mobilization^17^,^18^,^19^.

In contrast to mIgM and mIgD, mIgGs contain cytoplasmic domains of considerable length. Experiments using genetically modified mice demonstrated that the cytoplasmic tail of mIgG1 plays a key role in the activation and maintenance of mIgG1-expressing memory B cells^20^,^21^,^22^. Recently, two distinct signal-amplifying peptide motifs that differ in their proposed mode of operation were identified in the cytoplasmic domains of mammalian mIgG isotypes^6^,^7^,^23^. An SSVV (single-letter code for amino acids) peptide motif that is found within the 10 membrane-proximal amino acids of mammalian mIgG tails but not in mIgE tails was suggested to enhance signalling by constitutively recruiting the PDZ domain-containing scaffold protein SAP97 (ref. 23). The second motif represents a tyrosine phosphorylation-dependent interaction site for SH2 domain-containing effector molecules that is evolutionarily conserved in mIgG and mIgE isotypes^6^. On phosphorylation, this immunoglobulin tail tyrosine (ITT) motif recruits the adaptor protein growth factor receptor-bound 2 (Grb2) into the BCR signalosome to amplify antigen-induced Ca^2+^ mobilization and cellular proliferation. Inactivation of the ITT motif abolished enhanced mIgG-BCR signalling resulting in an mIgM-BCR-like signalling profile^6^.

The ubiquitous adaptor protein Grb2 has multiple roles in B cells. Initially, it was recognized as part of an inhibitory signal complex together with the adaptor protein Dok-3 that restricts BCR-induced Ca^2+^ mobilization in mIgM-expressing cells^17^,^24^,^25^,^26^. Furthermore, it may be involved in signalling of the negative regulatory coreceptor CD22^27^ and chemokine receptors in germinal centre B cells^28^. However, recently we provided evidence that in mIgG-expressing cells Grb2 promotes B-cell activation by supporting the mobilization of Ca^2+^ on binding to the phosphorylated ITT. Yet, the microanatomy of this signal-amplifying mechanism that underlies the robust reactivation of IgG-switched memory B cells remained unclear. Here, we demonstrate that the ITT motif represents the principle signal amplification device of memory-type BCRs in higher vertebrates. We find that PTKs of different families are involved in ITT-mediated signal amplification. Whereas Syk catalyses the phosphorylation of the ITT to allow for the recruitment of Grb2, Btk is involved in subsequent amplification of PLC-γ activity and sustained Ca^2+^ mobilization. Collectively, these mechanisms greatly increase the antigen sensitivity of isotype-switched B cells compared with cells that solely rely on ITAM-induced activation signals, which can explain their preferential reactivation in secondary immune responses.

## Results

### The ITT is the main signal amplifier of memory-type BCRs

**Figure 1:**
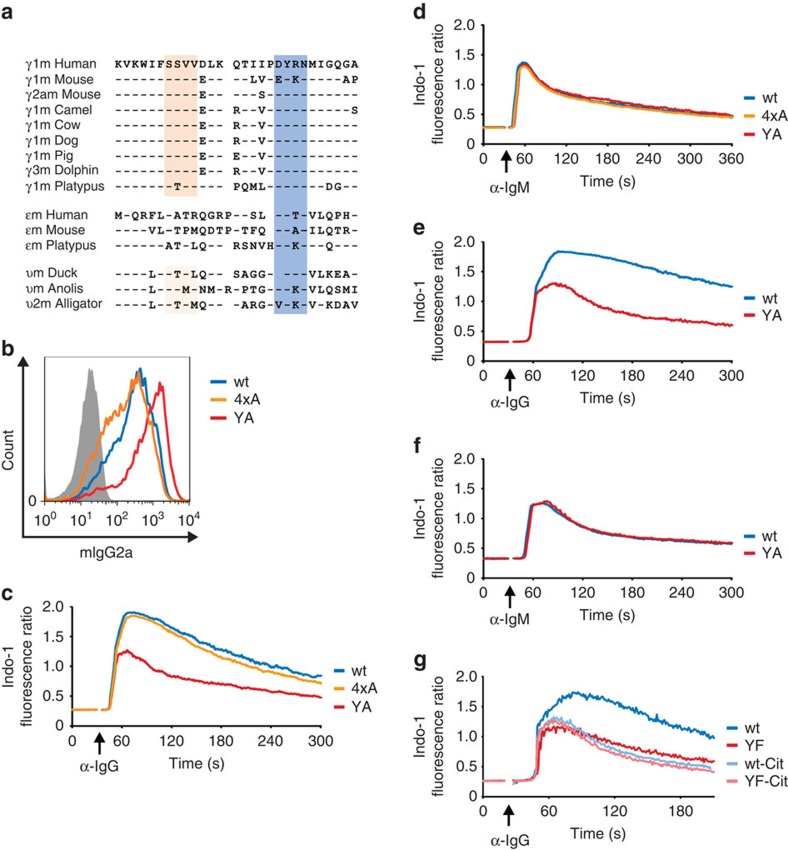
The ITT is the principal signal amplification device of mIgG-containing BCRs. (**a**) Amino-acid sequence alignment (single-letter code for amino acids) of cytoplasmic tails of γm (mIgG), εm (mIgE) or υm (mIgY) immunoglobulin heavy chains of the indicated species. The putative PDZ domain binding SSVV motif is highlighted in orange, the ITT motif in blue. (**b**) Surface expression of the indicated mIgG2a-BCR variants on DG75 B cells. (**c**,**d**) Ca^2+^ mobilization kinetics on stimulation with F(ab′)_2_ fragments to IgG (20 μg ml^−1^) (**c**) and IgM (20 μg ml^−1^) (**d**) were analysed in the presence of 1 mM extracellular CaCl_2_. (**e**,**f**) DG75 cells expressing chimeric mIgG/Y molecules were analysed as before. (**g**) C-terminally Citrine-tagged mIgG2a variants (wt, light blue line and ITT-mutant (YF) light red line) were expressed in DG75 cells, and anti-IgG-induced Ca^2+^ mobilization kinetics were analysed as before. Non-tagged γ2am versions (dark blue and dark red lines) served as controls. Data are representative of at least three independent experiments.

The cytoplasmic domains of mIgG isotypes have been reported to contain two distinct signal-amplifying peptide motifs. One comprises the amino acids SSVV^23^ and is evolutionarily conserved within the 10 membrane-proximal amino acids of mammalian mIgG tails but not in mIgE and only to some extent in mIgY tails of birds and reptiles (Fig. 1a). By contrast, the ITT motif is conserved not only in mIgGs but also in mIgE as well as in primordial mIgY molecules (Fig. 1a). To assess the individual contribution of the different peptide motifs for enhanced mIgG-BCR signalling, we generated alanine mutants of the SSVV (4xA) and ITT (YA) motifs and compared their Ca^2+^ mobilization capacities with that of wild-type BCRs. Surface expression of the different mIgG-BCR variants in the human B-cell line DG75 was similar, even though the YA variant was somewhat more abundant on the cell surface than wild type and 4xA-mutant mIgG (Fig. 1b). As shown in Fig. 1c, inactivation of the SSVV motif (orange line) had little effect on mIgG-BCR-mediated signal amplification as opposed to the robust drop caused by inactivation of the ITT (red line). As control, all cells were stimulated via their endogenous mIgM-BCRs to verify equal ITAM-mediated Ca^2+^ signalling capacities of the transfectants (Fig. 1d). To test whether the ITT motifs in primordial mIgY molecules of reptiles and birds are functionally conserved, we generated chimeric mIgG/Y molecules in which the cytoplasmic domain of mouse γ2am was replaced with that of υm from the lizard *Anolis carolinensis*. Stimulation of this chimeric receptor caused a very strong and sustained Ca^2+^ response, which was reverted to an mIgM-BCR-like response on inactivation of the Anolis ITT (Fig. 1e). As before, the integrity of the Ca^2+^ pathway in the transfectants was verified through stimulation of their endogenous mIgM-BCRs (Fig. 1f). These results show that the ITT is the principal signal amplification device of memory-type BCRs in higher vertebrates.

However, microscopic analyses of mIgG-molecules that were fused to a fluorescent protein tag at their C termini (that is, intracellular) suggested that the ITT motif plays a minor part in enhancing Ca^2+^ mobilization^7^. In search of an explanation for these conflicting results, we generated C-terminally Citrine-tagged variants of mIgG and tested whether such intracellular fluorophore tagging of mIgG tails influences their signalling behaviour. Indeed, the fluorophore tag completely abrogated ITT-mediated amplification of Ca^2+^ mobilization (Fig. 1g), which might be caused by sterical hindrance of SH2 domain-mediated binding to the phospho‐ITT. On balance, the ITT motif, which appeared earlier in vertebrate evolution than the SSVV motif and furthermore has been conserved in mIgY, mIgG and mIgE molecules, appears to be the major signal-boosting element of memory-type BCRs.

### The ITT is phosphorylated by the BCR transducer kinase Syk

**Figure 2:**
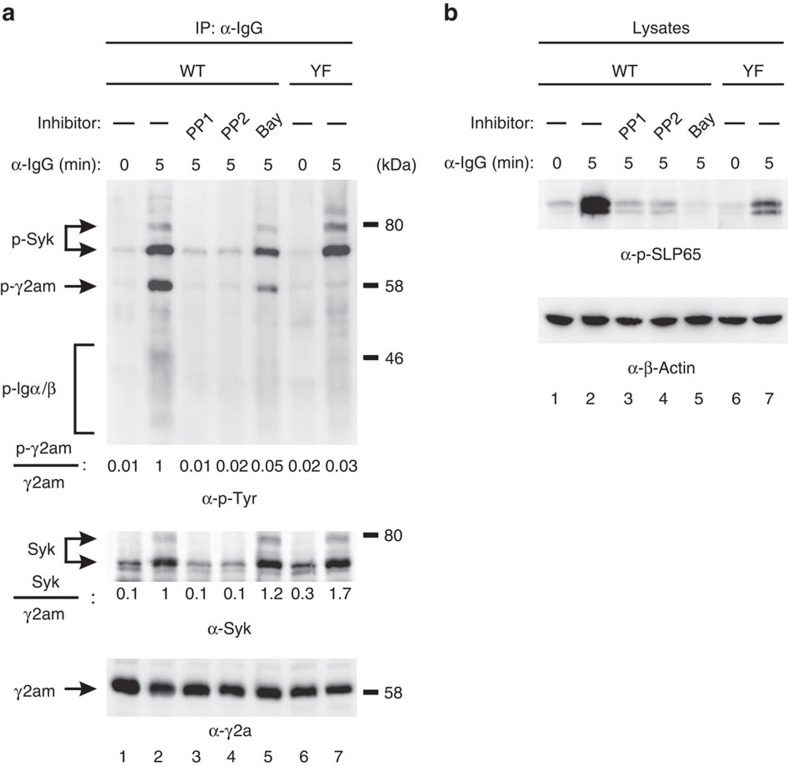
The ITT is phosphorylated by BCR-bound Syk. Ramos cells expressing either wild type (WT) or ITT-mutant (YF) mIgG2a were left untreated or pre-incubated with the Src kinase inhibitors PP1 or PP2 or the Syk inhibitor BAY61-3,606 for 30 min at room temperature. Subsequently, cells were left unstimulated (0) or stimulated for 5 min with polyclonal goat anti-mouse IgG antibodies, lysed and used for affinity purifications of mIgG2a-BCRs with protein A/G agarose beads (**a**). Purified proteins were successively analysed with antibodies to phosphotyrosine (α-p-Tyr, upper panel), Syk (middle panel) and γ2a (lower panel). Protein band intensities were determined and normalized as indicated on the left. (**b**) Aliquots of the same lysates used in (**a**) were analysed with anti-phospho-SLP65 (Y96) (upper panel) and anti-β-Actin (lower panel) as loading control. Data are representative of three independent experiments.

To better understand the molecular microanatomy of ITT-mediated signal amplification, we addressed the question as to which kinase acts upstream of the ITT. We previously showed that efficient phosphorylation of the ITT requires its incorporation into the BCR complex^6^. To test which of the two BCR-bound PTKs, Src-family kinases (SFKs) or Syk, phosphorylates the ITT, we analysed its phosphorylation in mIgG-expressing human B cells that were pre-incubated with either the SFK inhibitors PP1 or PP2 or the Syk inhibitor BAY61-3606 (Fig. 2 and Supplementary Fig. 1). Treatment of the cells with either PP1 or PP2 reduced not only BCR-induced tyrosine phosphorylation of the ITT to background levels but also that of Igα/β, Syk (Fig. 2a upper panel, lanes 3 and 4) and SLP65 (Fig. 2b), consistent with the common view that SFKs are the most upstream PTKs in the BCR signalling cascade. Notably, inhibition of SFKs disrupted the association between the BCR and Syk (Fig. 2a, middle panel, lanes 3 and 4), leaving open the possibility that recruitment of Syk to the BCR was required for ITT phosphorylation. By contrast, pharmacological inhibition of Syk did not interfere with its association with Igα/β (Fig. 2a, middle panel, lane 5), yet phosphorylation of the ITT was almost completely extinguished (lane 5, upper panel). The effectiveness of Syk inhibition was demonstrated by the absence of BCR-induced SLP65 phosphorylation in the cells (Fig. 2b, lane 5). Thus, Syk appears to be the kinase that directly phosphorylates the ITT, whereas SFKs are essential for phosphorylation of Igα/β and hence activation of Syk.

### The adaptor protein Grb2 is an essential ITT effector

**Figure 3:**
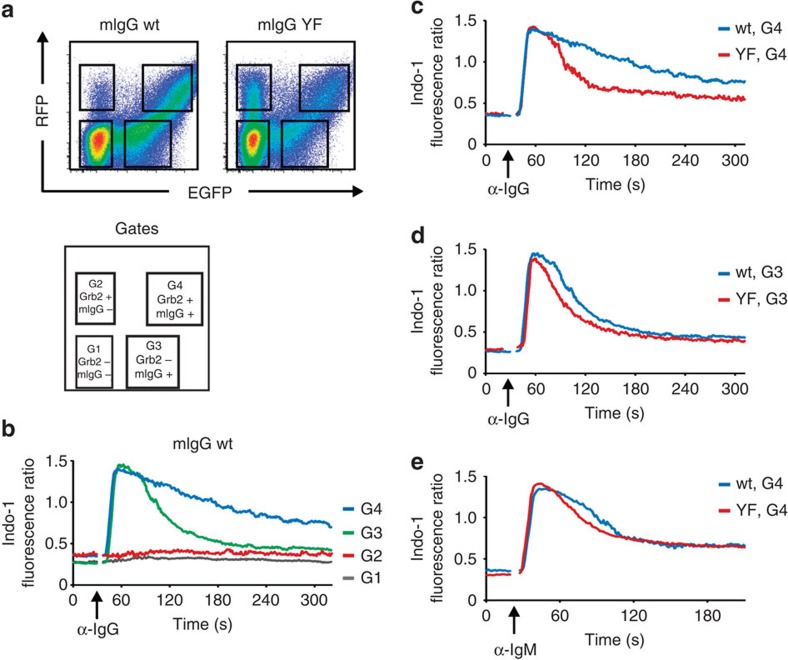
ITT signalling requires expression of Grb2. (**a**) Splenic mouse B cells deficient for Grb2 were retrovirally transfected with expression vectors encoding mouse γ2am (wt or YF) together with IRES-driven EGFP and Grb2 along with IRES-driven RFP. The resulting populations in the different gates (G1–G4) are shown on the right. The cells were stimulated with F(ab′)_2_ fragments against IgG (α-IgG, indicated by an arrow, 20 μg ml^−1^) (**b**–**d**) or IgM (α-IgM, 20 μg ml^−1^) (**e**). (**b**) Ca^2+^ mobilization kinetics of cells from all gates transfected with wild-type γ2am. (**c**,**d**) Ca^2+^ mobilization kinetics of cells expressing either wild type (wt, blue curves) or YF-mutant (red curves) mIgG2a-BCRs from gate G4 (**c**) and G3 (**d**) on anti-IgG stimulation or G4 on anti-IgM stimulation (**e**). Ca^2+^ mobilization was recorded in the presence of 1 mM extracellular CaCl_2_. Data are representative of three independent experiments.

A direct binding partner of the phosphorylated ITT is the ubiquitous adaptor protein Grb2 (ref. 6). To test whether Grb2 expression was required for ITT-mediated amplification of proximal BCR signalling events in a genetic model system, we retrovirally expressed either wild type (wt) or tyrosine to phenylalanine-mutant (YF) mIgG along with EGFP in murine splenic B cells from mice with a B-cell-specific deletion of the *Grb2* gene^26^. Measurement of mIgG-BCR-induced Ca^2+^ mobilization showed that the signal-amplifying effect of the ITT was abolished in Grb2-deficient B cells (Supplementary Fig. 2). Next, we doubly transduced Grb2-deficient primary B cells with wt or YF-mutant γ2am together with a second retroviral vector that reconstituted the cells with Grb2 along with IRES-driven expression of a red fluorescent protein (RFP). This transduction strategy gave rise to four populations of cells that could be distinguished according to the fluorescence of EGFP and/or RFP (Fig. 3a). B cells expressing wt mIgG together with Grb2 (gate G4 in Fig. 3a) showed a strong and long-lasting BCR-induced Ca^2+^ flux (Fig. 3b, blue curve) compared with the transient Ca^2+^ release in cells expressing mIgG but no Grb2 (gate G3, Fig. 3b, green curve). Cells that were EGFP-negative (gates G1 and G2) and hence did not express mIgG did not respond to mIgG-BCR stimulation (red and grey lines). Comparison of cells expressing either wt or YF-mutant mIgG-BCRs along with Grb2 from gate G4 (Fig. 3c) and those from gate G3 (Fig. 3d) showed that both the presence of a functional ITT as well as the expression of Grb2 were needed for enhanced mIgG-BCR-mediated Ca^2+^ signalling. Stimulation of endogenous mIgM-BCRs showed that transduced cells from G4 had the same ITAM-mediated signalling capacities (Fig. 3e). Similar results were obtained in the human B-cell line DG75 in which we inactivated the *GRB2* gene by gene targeting (see Supplementary Figs 3 and 4). These genetic experiments identify the universal adaptor protein Grb2 as essential component for improved signalling of mIgG-BCRs, which is consistent with the dispensability of the putative SSVV motif in that process.

### IgG-switched memory B cells require Grb2 for reactivation

**Figure 4:**
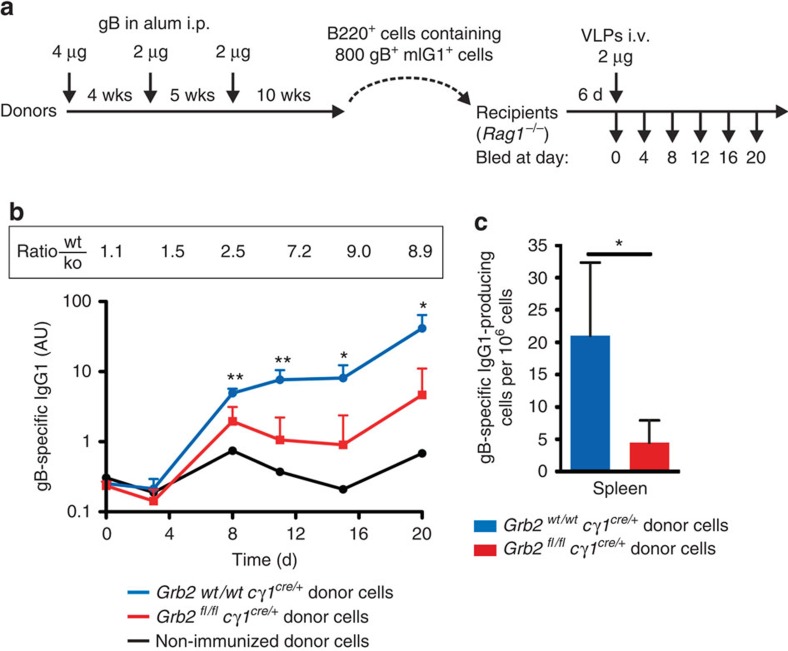
T-cell-independent reactivation of IgG-switched memory B cells requires Grb2. (**a**) Experimental outline. *Grb2*^*wt/wt*^*Cγ1*^*cre/+*^ (*n*=3) and *Grb2*^*fl/fl*^*Cγ1*^*cre/+*^ mice (*n*=5) were repeatedly immunized with purified gB with aluminium hydroxide (alum) at the indicated time points. Splenic B cells were purified 70 days after third immunization by complement-mediated T-cell lysis and anti-CD19 magnetic bead separation. CD19-positive cells containing 800 memory B cells each (identified as B220+, IgG1+, gB+) were transferred intravenously into *Rag1*^−/−^ recipient mice and challenged 6 days later by an intravenous injection of 2 μg virus-like particles (VLPs) of human CMV in PBS. (**b**) gB-specific IgG1 titres were measured by ELISA. (**c**) ELISPOT for gB-specific IgG1-secreting cells was performed at day 43 after VLP challenge. Error bars represent mean+s.d. of at least three analyses; Student’s *t*-test was used. *P<0.05 **P<0.01.

Having established that the ITT of memory-type BCRs and the adaptor protein Grb2 constitute a functional duo, we tested whether Grb2 is required for the reactivation of IgG-switched memory B cells *in vivo* using a genetic model system. To this end, we generated mice in which the *Grb2* gene gets deleted in germinal centre (GC) B cells by crossing *Grb2*^*fl/fl*^ mice^26^ with Cγ1^cre^ mice^29^. In the resulting *Grb2*^*fl/fl*^ *C*γ*1*^*cre/+*^ animals, the expression of Grb2 is efficiently shut off in GC B cells (Supplementary Fig. 5). The mice were repeatedly immunized with purified glycoprotein B (gB) of human cytomegalovirus (hCMV) to induce formation of an antigen-specific mIgG1-positive memory B-cell population. Subsequently, gB-specific IgG1+ memory B cells were isolated, transferred into *Rag1*-deficient recipient mice and challenged 6 days later with gB-containing virus-like particles (VLPs) (Fig. 4a). The usage of *Rag1*-deficient recipients allowed us to study the reactivation of the transferred memory B cells in the absence of T-cell help. The gB-specific IgG1 responses were determined for 20 days, which revealed that wild-type memory B cells gave rise to up to ninefold higher IgG1 titres as compared with Grb2-deficient cells (Fig. 4b). This difference was furthermore reflected by a substantially higher number of IgG1-secreting plasma cells in the spleens of the recipient mice 43 days after the challenge (Fig. 4c). Similar results were obtained when *Grb2* was deleted in all B cells using *mb1*-cre deleter mice (Supplementary Fig. 6). Taken together, our results show that Grb2 is essential for enhanced signalling of ITT-containing BCR isotypes, which greatly improves their T-cell-independent reactivation on antigen reencounter *in vivo*.

### The N-terminal Grb2 SH3 domain mediates ITT signalling

**Figure 5:**
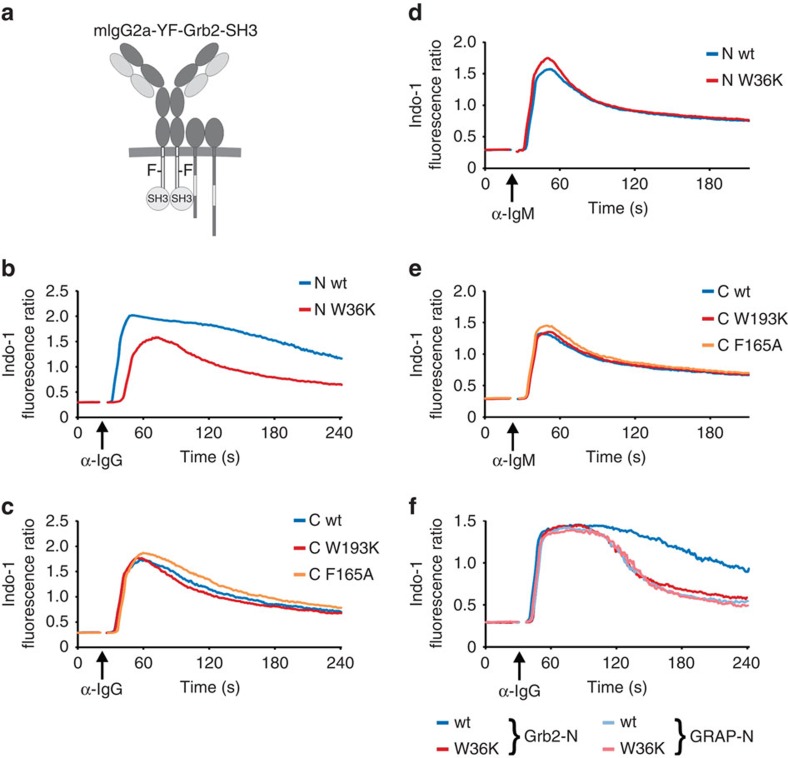
The N-terminal SH3 domain of Grb2 mediates enhanced Ca^2+^ signalling downstream of the ITT. (**a**) Schematic illustration of chimeric receptor variants used in this study. Chimeric constructs consisting of mouse YF-mutant γ2am fused to the N-terminal (**b**) or C-terminal (**c**) SH3 domains of Grb2 were retrovirally expressed in DG75 B cells. The SH3 domains were either wild type (wt, blue curves) or inactivated by tryptophan to lysine (W36K and W193K, respectively, red curves) or a phenylalanine to alanine (F165A, orange curve) mutations. Cells were either stimulated with anti-IgG or anti-IgM F(ab′)_2_ fragments (indicated by arrows) and Ca^2+^ mobilization was recorded in the presence of 1 mM extracellular CaCl_2_. (**d**) Chimeric γ2am-YF-chimeras containing the N-terminal SH3 domain of either Grb2 (wt: dark blue line, W36K: dark red line) or GRAP (wt: light blue line, W36K: light red line) were retrovirally expressed in murine splenic B cells and analysed as before. Data are representative of four independent experiments.

The adaptor protein Grb2 is composed of a central SH2 domain that is flanked by an N-terminal and a C-terminal SH3 domain. Both SH3 domains were reported to have exclusive and overlapping interaction partners^30^. To investigate the role of the individual Grb2 SH3 domains in an mIgG-BCR-specific manner, we generated chimeric mIgG-molecules in which we fused the respective Grb2 SH3 domains to the cytoplasmic tail of YF-mutant γ2am (see Fig. 5a). As controls we used inactivated variants of these domains (W36K, W193K or F165A, respectively). All constructs were expressed in similar amounts on the surface of the human B-cell line DG75 (data not shown). Analysis of anti-IgG-induced Ca^2+^ mobilization showed that in the context of such chimeric BCRs the N-terminal (Fig. 5b) but not the C-terminal (Fig. 5c) SH3 domain of Grb2 was able to replace the ITT motif. As control, all cells were stimulated via their endogenous mIgM-BCRs to demonstrate equal Ca^2+^ mobilization capacities of the transductants (Fig. 5d,e).

To test whether the mIgG2a-Grb2N-SH3 chimera still required expression of endogenous Grb2, we expressed this receptor in our *GRB2*-deficient DG75 B cells. Clearly, the enhanced signalling capacity of the chimera did not require the presence of Grb2 (Supplementary Fig. 7), showing that selective incorporation of the N-terminal SH3 domain of Grb2 into the YF-mutant mIgG-BCR complex was sufficient to restore the Ca^2+^-promoting effect of the ITT.

Besides Grb2 we identified the Grb2-related adaptor protein (GRAP) as a second direct ITT-binding protein (Supplementary Fig. 8). To test whether GRAP can fulfil a similar role in ITT signalling as Grb2, we generated chimeras of mIgG2a and the individual SH3 domains of GRAP. However, in contrast to the N-terminal SH3 domain of Grb2 both SH3 domains of GRAP failed to boost BCR-induced Ca^2+^ mobilization in the context of chimeric mIgG2a-BCRs in primary mouse B cells (Fig. 5f and data not shown). These results show that the N-terminal SH3 domain of Grb2 specifically interacts with positive regulators of Ca^2+^ mobilization in B cells.

**Figure 6:**
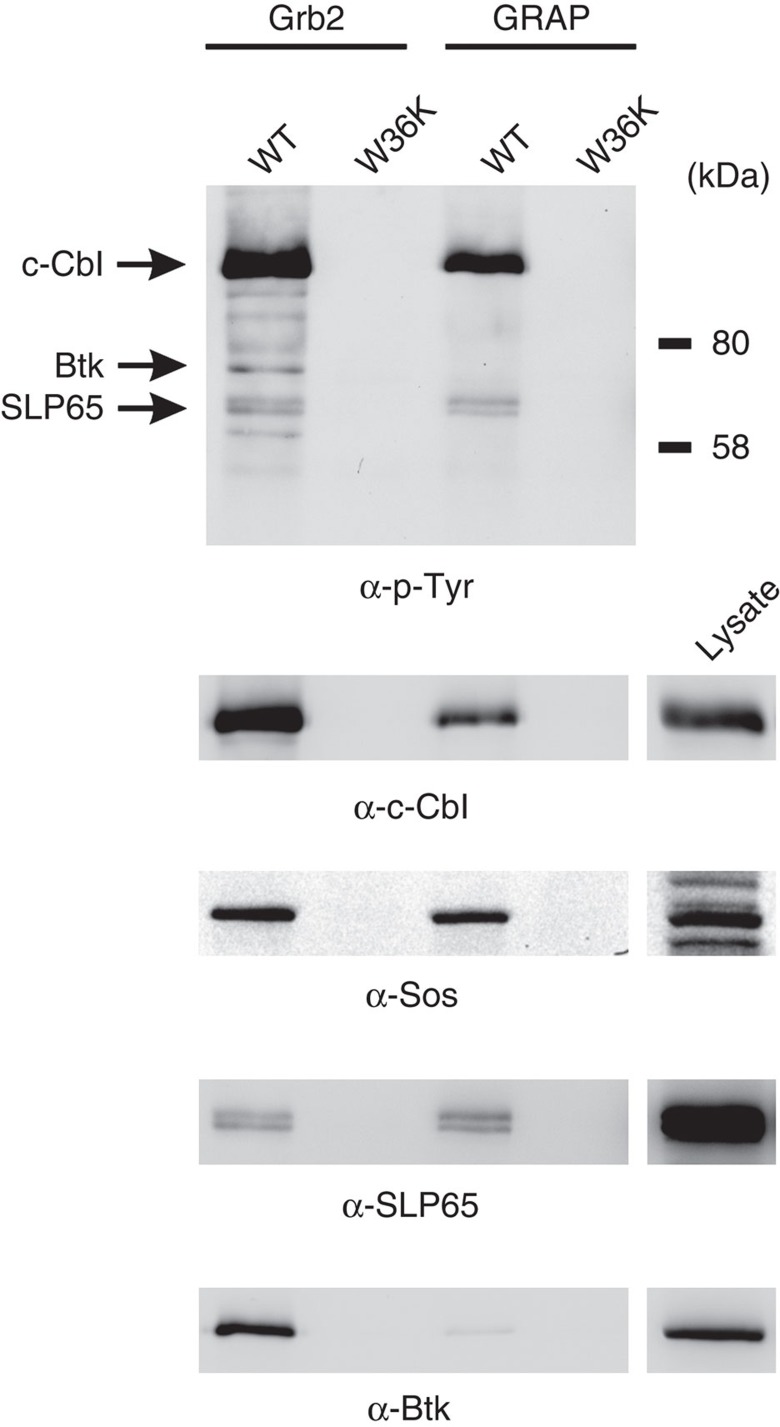
Interaction partners of the N-terminal Grb2 SH3 domain in B cells. DG75 B cells were stimulated via their BCR for 3 min to induce tyrosine phosphorylation of signalling proteins. Subsequently, lysates of these cells were used for affinity purifications with GST-coupled N-terminal SH3 domains of Grb2 and GRAP. Inactivated (W36K) variants were used as controls. Purified proteins were analysed by immunoblotting with anti-phosphotyrosine (α-p-Tyr) antibodies (upper panel) and antibodies to c-Cbl, Sos, SLP65 and Btk as indicated. To assess the efficiency of affinity purification, signals of lysates (equivalent to 2% of input lysates) are shown on the right. Data are representative of four independent experiments.

To identify candidate proteins that may mediate the enhanced Ca^2+^ signalling downstream of Grb2, we performed affinity purifications with the N-terminal SH3 domains of Grb2 and GRAP from lysates of BCR-activated human B cells and compared their interaction partners by immunoblot analyses (Fig. 6 and Supplementary Fig. 9). The most prominent tyrosine-phosphorylated protein that was purified with both SH3 domains co-migrated with the c-Cbl (2nd panel from the top) and Cbl-b (not shown) ubiquitin ligases. Furthermore, both SH3 domains associated with the guanine nucleotide exchange factor Sos and weakly bound to SLP65. As the GRAP N-SH3 domain could not functionally replace the N-SH3 of Grb2 (see Fig. 5), these common interaction partners were most likely not involved in ITT-mediated Ca^2+^ signalling. In searching for specific interaction partners of the N-SH3 of Grb2 that are known to regulate Ca^2+^ mobilization in B cells, we identified the PTK Btk as a novel interaction partner of Grb2 but not GRAP (Fig. 6, bottom panel). Noteworthy, we did not observe associations of the N-terminal Grb2 SH3 domain with Src- or Syk-family PTKs nor with PLC-γ isoforms (data not shown). Given the well-documented role of Btk in BCR-induced Ca^2+^ mobilization, we investigated its putative function in ITT signalling in more detail.

### Grb2 amplifies signalling of memory-type BCRs via Btk

**Figure 7:**
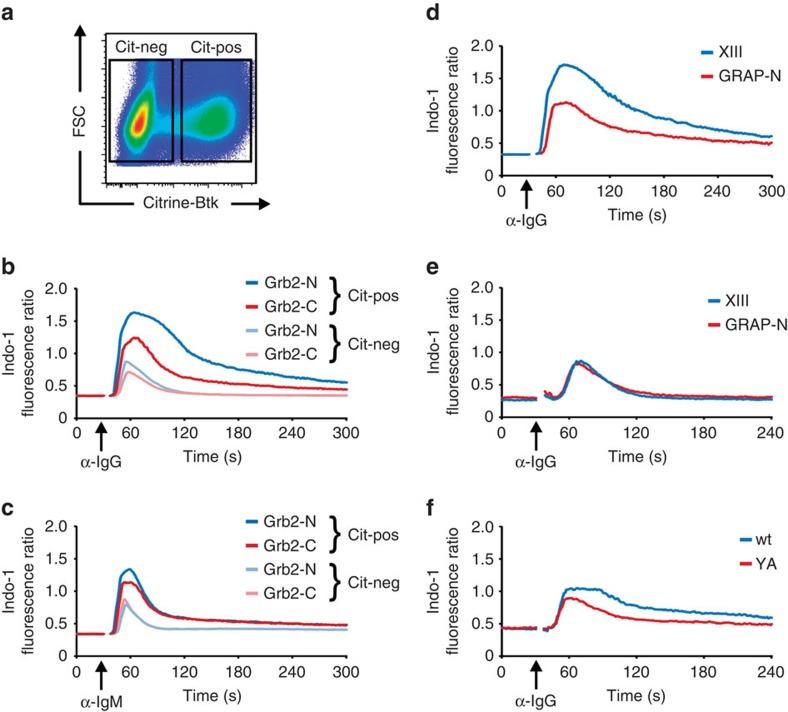
Active amplification of Ca^2+^ signalling by Grb2 requires Btk. *BTK*-deficient DG75 B cells were retrovirally transduced to express chimeric γ2am-YF molecules containing either the N-terminal (blue lines) or C-terminal (red lines) SH3 domains of Grb2. Subsequently, the cells were retrovirally transfected with Citrine-tagged Btk, resulting in two populations that were either Btk-negative (Cit-neg) or Btk-positive (Cit-pos) (**a**). BCR-induced Ca^2+^ mobilization was analysed in all populations on stimulation with polyclonal F(ab′)_2_ fragments to IgG (**b**) or IgM (**c**). (**d**) Wild-type DG75 cells expressing chimeric γ2am-YF molecules containing either the N-terminal SH3 domain of GRAP (red line) or a variant thereof (XIII) having three amino-acid substitutions at positions three, four and five (S3A, V4I and L6K, blue line) were analysed as before. (**e**) The same chimeric receptors as in (**d**) were analysed in BTK-deficient DG75 cells. (**f**) *BTK*-deficient DG75 cells were retrovirally transfected with wild type (wt, blue line) or tyrosine to alanine mutant (YA, red line) γ2am-encoding expression vectors and stimulated as before. Data are representative of at least three independent experiments.

To test whether recruitment of Btk to the mIgG-BCR represents an essential ITT effector mechanism, we generated a *BTK*-deficient variant of DG75 cells by homologous recombination (for details see Supplementary Fig. 10) and expressed the chimeric mIgG2a-YF-Grb2-SH3 constructs in these *BTK*-deficient cells. Indeed, the signal-amplifying effect of the N-terminal Grb2 SH3 domain was abolished in the absence of Btk expression (Supplementary Fig. 11). Next, we retrovirally reconstituted the cells with a Citrine-tagged variant of wild-type Btk. This strategy gave rise to two populations of cells that were either Btk-negative (Fig. 7a, Cit-neg) or Btk-positive (Cit-pos) and hence could be differentiated during Ca^2+^ flux analysis by gating. In contrast to the situation in Citrine-positive cells (Fig. 7b, Cit-pos) and wild-type cells (see Fig. 5), the N-terminal SH3 domain of Grb2 did not amplify Ca^2+^ mobilization compared with the C-terminal Grb2 SH3 domain in the absence of Btk (Fig. 7b, Cit-neg). As control the different transfectants were stimulated via their endogenous mIgM-BCRs (Fig. 7c). To test whether a direct interaction between Grb2 and Btk was required for this Ca^2+^-promoting circuit, we mapped and inactivated the Grb2-binding site in Btk (data not shown). However, inactivation of the respective proline-rich motif in Btk caused an almost complete loss of function of Btk and hence did not provide conclusive evidence for the need of a direct interaction between the two proteins in this process (data not shown, manuscript in preparation). To circumvent this drawback, we mapped the amino-acid residues in the N-terminal SH3 domain of Grb2 that are necessary for its interaction with Btk by constructing a series of Grb2/GRAP chimeric SH3 domains (see Supplementary Figure 12). With this approach we identified three amino acids at the very N-terminus of the Grb2 N-SH3 that restore binding to Btk if transplanted into the GRAP N-SH3. The respective chimeric SH3 domain (referred to as ‘XIII’) was fused to ITT-mutant mIgG2a as before and transfected into DG75 B cells. Indeed, this Btk-binding variant of the GRAP N-SH3 amplified BCR-induced Ca^2+^ mobilization as well as the Grb2 N-SH3 domain (Fig. 7d). However, when we expressed this chimeric mIgG-BCR in *BTK*-deficient cells, the Ca^2+^-amplifying effect of the XIII SH3 domain variant was abolished (Fig. 7e).

To test the signalling capacity of the intact ITT in the absence of Btk, we expressed wild type and ITT-mutant mIgG-BCRs in our *BTK*-deficient DG75 cells. In this experimental setting, the ITT in wild-type mIgG still evoked a moderately increased Ca^2+^ signal compared with ITT-mutant mIgG (Fig. 7f), suggesting that additional Ca^2+^-amplifying mechanisms operate in parallel to recruitment of Btk to ITT-bound Grb2.

### ITT signalling increases the antigen sensitivity of mIgG-BCRs

**Figure 8:**
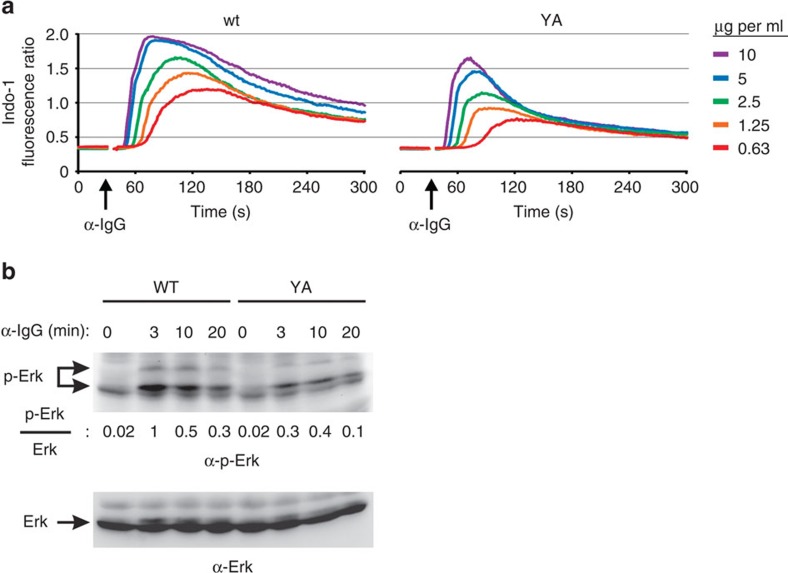
The ITT increases the sensitivity of mIgG-BCRs for antigen. (**a**) Ca^2+^ mobilization kinetics of DG75 cells expressing wild type (wt) or ITT-mutant (YA) mIgG2a-BCRs on stimulation with decreasing amounts of anti-IgG F(ab′)_2_ fragments. Data are representative of two independent experiments. (**b**) The same cells were stimulated with 0.63 μg ml^−1^ anti-IgG F(ab′)_2_ fragments for the indicated times, and activation of Erk was determined by immunoblot analysis with antibodies to phosphorylated Erk (upper panel). Anti-Erk served as a loading control (lower panel). The ratio of phosphorylated (p-Erk) to total Erk is given. Data are representative of three independent experiments.

Our data show that ITT/Grb2-mediated signal amplification has a pivotal function in reactivation of mIgG-BCR-expressing memory B cells *in vivo* (see Fig. 4). To test whether this improved cellular activation is reflected by a heightened sensitivity of mIgG-BCRs compared with ITT-less BCR isotypes, we titrated stimulating anti-IgG F(ab’)_2_ fragments and used the measurement of Ca^2+^ mobilization kinetics as a read-out for the effectiveness of BCR stimulation. The results showed that wild-type mIgG-BCRs needed ~4 times less stimulating agent to induce a Ca^2+^ peak comparable to that of ITT-mutant BCRs (Fig. 8a, compare green curve of wt mIgG (2.5 μg ml^−1^) with purple curve of YA-mutant mIgG (10 μg ml^−1^)). Notably, stimulation with as little as 0.63 μg ml^−1^ anti-IgG F(ab′)_2_ fragments induced a longer lasting Ca^2+^ response in wild-type mIgG-expressing cells than optimal stimulation of ITT-mutant mIgG. Furthermore, we tested the activation of the Erk MAP kinase pathway in the cells under suboptimal stimulation conditions. In accordance with the enhanced Ca^2+^ flux, wild-type mIgG-BCRs induced a more robust and longer lasting phosphorylation of Erk proteins than ITT-mutant BCRs (Fig. 8b and Supplementary Fig. 13). Hence, signal amplification by the ITT/Grb2/Btk signalling circuit drastically lowers the amount of antigen needed to deliver an activation signal into the cell, which can explain why memory B cells with an intact ITT/Grb2 module have an advantage over cells that lack either component to get reactivated by small amounts of antigen.

## Discussion

A hallmark of secondary immune responses is the switch in antibody isotype production from IgM to IgG. This observation goes back roughly half a century^5^ and has been refined more recently in elegant *in vivo* studies^3^,^4^. However, the molecular mechanisms that underlie the preferential production of IgG antibodies on antigen reencounter remain incompletely understood. Recently, we showed that the cytoplasmic domains of membrane-bound γ and ε Ig heavy chains contain a signal amplification motif that we termed the ITT^6^. In addition to the ITT, a putative PDZ domain-recruiting peptide motif containing the amino-acid sequence SSVV has been suggested to contribute to the improved responsiveness of mIgG-expressing memory B cells^23^. When comparing the two motifs in our cellular model systems, the SSVV motif had little effect on signal initiation by mIgG-BCRs and its role for IgG responses *in vivo* remains to be seen. However, a recent report demonstrated that the SSVV-binding protein SAP97 is dispensable for primary and secondary antibody responses as both processes were normal in immunodeficient recipient mice that had been repopulated with *Dlg-1*- (*SAP97*)-deficient fetal liver cells and hence lacked SAP97 expression in B and T cells^31^.

**Figure 9:**
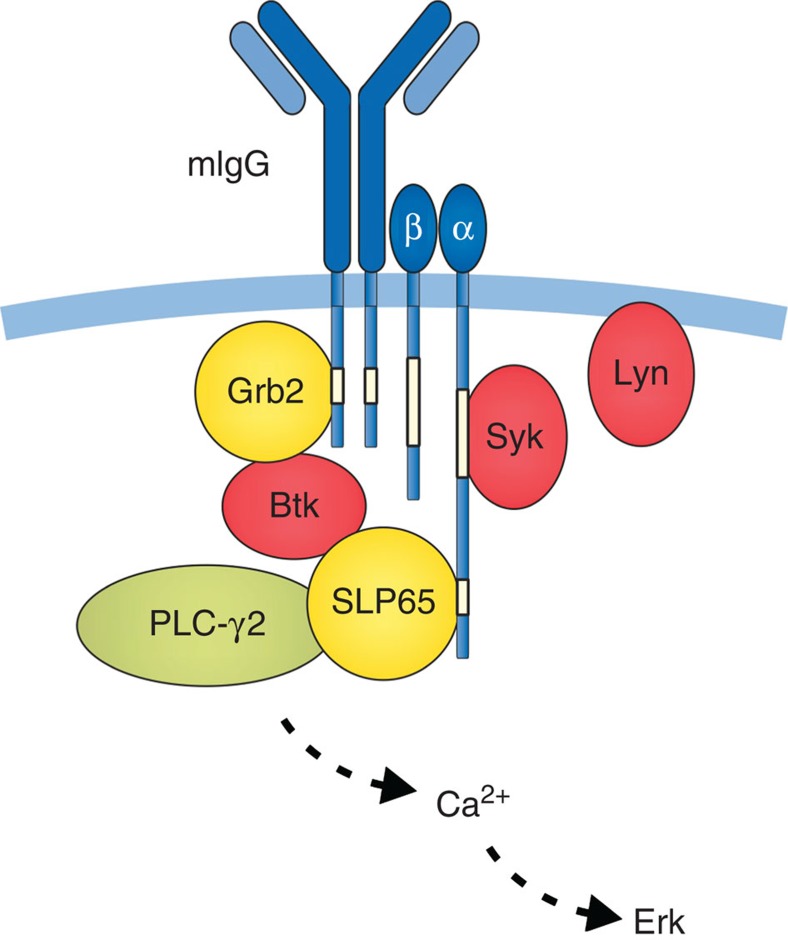
Amplification of mIgG-BCR signalling by the ITT/Grb2/Btk signalling module. On phosphorylation by ITAM-bound Syk, the ITT motifs (white boxes in cytoplasmic mIgG tails) provide docking sites for the ubiquitous adaptor protein Grb2. Grb2 in turn brings along Bruton’s tyrosine kinase (Btk) via a constitutive interaction that is mediated by the N-terminal SH3 domain of Grb2. The incorporation of Grb2/Btk into the BCR signalosome stabilizes the Ca^2+^ initiation complex consisting of Btk, the adaptor protein SLP65 (which in addition interacts with a non-ITAM tyrosine-phosphorylation motif in Igα) and phospholipase C-γ2 (PLC-γ2) at the activated receptor, and thereby lowers the threshold for activation of PLC-γ2 by Btk. This active signal amplification loop is complemented by a passive signal amplification that is brought about by sequestration of ITT-bound Grb2 from negative regulators of Ca^2+^ mobilization such as CD22 and Dok-3.

The ITT resembles tyrosine-based signalling motifs found in several other immune receptors like DAP10 and CD28 that have either stimulatory or costimulatory activity^32^. The ITT-like motifs in these receptors are mainly targets of Src PTKs^33^,^34^. However, in mIgG-BCRs the ITT is inevitably incorporated into the ITAM-containing microenvironment of the Igα/β signalling device where it gets phosphorylated by—most likely ITAM-bound—Syk (see Fig. 9). ITT phosphorylation creates a binding site for SH2 domain-containing effector proteins that confer costimulatory activity to mIgG-BCRs. Although the mIgG-ITT has a YXNM consensus motif that—on paper—predisposes for binding to both Grb2 adaptors via YXN and p85 subunits of PI3K via YXXM, only Grb2 family adaptors could be detected as direct interaction partners (ref. 6 and this study). In the present work, these biochemical findings are corroborated by genetic model systems showing that Grb2 is essential for enhanced signalling of mIgG-BCRs as well as the efficient reactivation of IgG-switched memory B cells by virus-like particles and subsequent production of IgG antibodies *in vivo*. A key role for Grb2 recruitment is furthermore reflected by the strict evolutionary conservation of the YXN core (but not YXXM) in all known vertebrate ITTs. Together, the available data from biochemical and genetic *in vitro* and *in vivo* model systems substantiate the importance of the mIgG tail and its ITT-Grb2 signalling module for the vigorous nature of secondary IgG antibody responses. However, the role of Btk in that process appears less clear. Humans with *BTK*-deficiency have very low B-cell numbers and hence have very low antibody titres of all isotypes. Secondary antibody responses in mice with compromised Btk function are variously affected, which might be caused by the usage of different mouse strains as well as divergent immunization protocols^35^,^36^,^37^. Furthermore, in mice—as opposed to humans—the Btk-related PTK Tec can compensate for loss of Btk in B cells^37^. Hence, it appears possible that in mouse B cells Tec complements Btk in the ITT pathway, which would explain the moderately affected production of IgG antibodies in mice lacking functional Btk. Furthermore, even in our human B-cell system the ITT still provoked a moderately enhanced Ca^2+^ signal in the absence of Btk, showing that further mechanisms contribute to ITT-based signal amplification. Noteworthy, sequestration of Grb2 from negative regulators of Ca^2+^ mobilization in B cells like Dok-3 and CD22 has been suggested as a way to improve the activation of PLC-γ and subsequent second messenger generation^17^,^24^. More specifically, Grb2 molecules that are hooked to phospho-ITTs are not available for association with phosphorylated negative regulators, resulting in a passive signal amplification that complements the active signal amplification by recruitment of Btk to memory-type BCRs. The importance of the Ca^2+^ initiation complex for IgG production and memory responses is furthermore reflected by the observation that maintenance and reactivation of IgG1-switched B cells require the expression of the Btk target PLC-γ2 (ref. 38).

To what extent BCR signals are required or even sufficient to induce terminal plasma cell differentiation is incompletely understood. Studies with a TD antigen of varying affinity showed that strong antigen recognition favours plasma cell generation^39^,^40^,^41^. As the strength of BCR signals rises with increasing affinity for antigen^42^, a picture emerges in which a B cell has two ways to increase its BCR signalling strength. First, by improving the affinity of its BCR for antigen and, second, by incorporating the signal-amplifying ITT into the BCR. Both modifications lead to enhanced intracellular Ca^2+^ mobilization, which seems to support the generation of plasma cells. Thus, B cells expressing ITT-containing BCRs have an advantage in contributing to antibody production over those cells expressing ITT-less BCRs if their antigen-affinities are the same. This may, at least in part, explain why IgG-switched memory B cells are preferentially reactivated by antigen over IgM-expressing memory cells^4^. However, additional mechanisms are likely to contribute to that process. For example, a recent study showed that certain transcription factors like Bach2 are downregulated specifically in mIgG1-expressing memory B cells, which may facilitate their rapid differentiation into plasma cells^43^.

In sum, class switching to IgG, IgE or IgY isotypes incorporates a signal-amplifying ‘costimulatory’ signalling device into memory-type BCRs that enhances the cell sensitivity for antigen and thus improves their competitiveness and reactivation in secondary immune reactions.

## Methods

### Cells and transfections

The human Burkitt lymphoma line DG75 was purchased from the German Collection of Microorganisms and Cell Cultures (DSMZ, Braunschweig, Germany). The cells were stably transfected with the cDNA of murine cationic amino-acid transporter 1 (*slc7a1*) to make them susceptible for infection with MMLV-based retrovirus particles and were selected with blasticidin S (10 μg ml^−1^). The resultant DG75EB cells were used in all experiments. DG75 cells deficient for *BTK* or *GRB2*, respectively, are described in detail in Supplementary Figs and were transfected wtih *slc7a1* alike. Ramos cells expressing murine γ2am variants were previously described^6^. All cells were cultured in RPMI1640+Glutamaxx (Biochrome) supplemented with 10% heat-inactivated FCS and antibiotics.

### Expression vectors

Retroviral expression vectors for mouse γ2am immunoglobulin heavy chains were previously described^6^. Mutations were introduced via PCR and verified by sequencing. Chimeric γ2am-SH3 domain constructs were generated by fusing the coding sequences of the respective SH3 domains to YF-mutant γ2am cDNA. Citrine-tagged γ2am molecules were generated in the same way. γ2am and Citrine were separated by the 12 amino-acid linker GGGAASGGGAAS (single-letter code for amino acids). All constructs were cloned into pMSCVpuro (Invitrogen). The cDNA of mouse Grb2 was cloned into pMiRFP containing an IRES-tagRFP (Evrogen) element. The cDNA of human Btk was cloned into pMSCVpuro-Citrine-C1 resulting in N-terminally Citrine-tagged Btk. cDNAs encoding chimeric SH3 domains were either chemically synthesized (Eurofins MWG Operon) or generated by PCR.

### Analysis of BCR-induced Ca^2+^ mobilization

For analysis of intracellular Ca^2+^ concentrations, cells were loaded with 1 μM Indo-1-AM (Molecular Probes) in RPMI containing 10% FCS and 0.015% pluronic acid (Molecular Probes) at 30 °C for 30 min under mild agitation. After washing, cells were suspended in Krebs Ringer solution composed of 10 mM HEPES (pH 7.0), 140 mM NaCl, 4 mM KCl, 1 mM MgCl_2_, 1 mM CaCl_2_ and 10 mM glucose. After monitoring basal Ca^2+^ concentrations for 30 s, cells were stimulated with the indicated reagents. The fluorescence ratio of Ca^2+^ bound (405 nm) versus Ca^2+^ unbound Indo-1 (530 nm) was monitored on an LSR II cytometer (Becton Dickinson). Data analysis was done using FlowJo software (TreeStar).

### Biochemical assays

For affinity purifications of mIgG2a-BCRs, 3 × 10^7^ cells were stimulated with 10 μg ml^−1^ goat anti-mouse IgG antibodies (SouthernBiotech) for 5 min at 37 °C, then spun down and lysed in ice-cold lysis buffer containing 50 mM Tris-HCl (pH 7.8), 137 mM NaCl, 0.5 mM EDTA, 1 mM sodium orthovanadate, 10% (w/v) glycerol, 1% (w/v) NP40 supplemented with a protease inhibitor cocktail containing AEBSF, Aprotinin, Bestatin, E-64, Leupeptin and EDTA (Sigma Aldrich, P2714). Insoluble material was pelleted at 20.000 × *g* for 10 min and cleared lysates were incubated with 30 μl of protein A/G PLUS agarose slurry (Santa Cruz biotechnology) for 1 h at 4 °C with gentle agitation. After extensive washing with lysis buffer, bound proteins were released by boiling with Laemmli buffer and separated on 10% polyacrylamide gels. Tyrosine kinase inhibitors PP1, PP2 and BAY61-3606 were from Calbiochem and were used at a final concentration of 30 μM. Affinity purifications with GST fusion proteins were done as described for purification of mIgG-BCRs, except that ~20 μg of fusion proteins bound to glutathione-sepharose beads were used. Antibodies to β-Actin (clone 13E5), Btk (D3H5), phospho-BLNK-Y96, phospho-Erk1/2 (E10) and phosphotyrosine (100) were from Cell Signaling. Anti-pan-Erk (clone 16), anti-Grb2 (clone 81) and anti-c-Cbl (clone 17) were from BD Transduction Laboratories. Anti-GRAP was from Abnova, anti-SLP65 from BAbCo and anti-Syk (4D10) from Santa Cruz. All antibodies were used at 1:1,000 dilutions for western blotting. Quantification of western blot band intensities was done using Gel-Pro Analyser software (Media Cybernetics). Polyclonal goat F(ab′)_2_ fragments for BCR stimulation were from Jackson ImmunoResearch.

### Mice

*Grb2*^*fl/fl*^ mice were described previously^26^. *Grb2*^*fl/fl*^ *mb1*^*cre/+*^ are on a mixed BALB/c/C57BL/6 background. The *Grb2*^*fl/fl*^ *C*γ*1*^*cre/+*^ were backcrossed for ten generations to C57BL/6 background. The Cγ1cre mice were a generous gift of S. Casola^29^. Animal experiments were approved by a local ethics committee of the government of Central Franconia (Regierung Mittelfranken).

### Retroviral transfections of primary mouse B cells

Splenocytes from 8- to 10-week-old female C57BL/6 mice (in house breeding) or corresponding B-cell-specific *grb2*-ko (*Grb2*^*fl/fl*^ *mb1*^*cre/+*^) mice^26^ were depleted of CD43-positive cells using anti-CD43 magnetic microbeads (Miltenyi Biotec). The remaining cells, which were >90% positive for CD19 and IgM, were incubated for 24 h with 10 μg per ml LPS (Sigma) in RPMI1640+Glutamaxx supplemented with 10% heat-inactivated FCS and antibiotics. Subsequently 2 × 10^6^ cells were infected with retrovirus-containing supernatants from Plat-E packaging cells supplemented with 3 μg ml^−1^ polybrene. Cells were used for assays 24 to 48 h after infection.

### Adoptive cell transfer

For memory cell transfer, 2- to 4–month- old male and female *Grb2*^fl/fl^ *mb1*^cre/+^ and corresponding *Grb2*^fl/fl^ *mb1*^+/+^ mice were immunized three times with the indicated amounts of hCMV glycoprotein B (gB, a gift from Sanofi Pasteur, Lyon, France). Eighty-three days after the last immunization, splenic cells were stained with B220-PE (RA3-6B2), IgG1-FITC (A85-1), IgG2a-FITC (R19-15), IgG2b-FITC (R12-3). B220+, IgG1+/IgG2a+/IgG2b+ cells were sorted, additionally stained with gB-Cy5 and sorted again for B220+, IgG1+/IgG2a+/IgG2b+, gB+ cells. Cells were sorted directly into tubes with *Rag1*^−/−^ splenic cells (as filler cells). Subsequently, the cell suspensions containing equal numbers of memory B cells for each genotype were adoptively transferred into 6-month-old male and female NK-cell depleted (depletion with anti-NK1.1 Ab from BioXcell) *Rag1*^−*/*−^ recipient mice (in house breeding) via tail vein injection. NK-cell depletion is necessary because of the mixed background of *Grb2*^*fl/fl*^ *mb1*^*cre/+*^ and *Grb2*^*fl/fl*^ *mb1*^*+/+*^ mice. After 6 days recipient mice were challenged by intravenous (i.v.) injection of 2 μg VLPs containing gB in their envelope and bled regularly. For memory cell transfer from *Grb2*^*fl/fl*^ *C*γ*1*^*cre/+*^ or *Grb2*^*wt/wt*^ *C*γ*1*^*cre/+*^ mice, two to four months old male and female animals were immunized three times with gB. 70 days after the last immunization CD19+ spleen cells were purified by magnetic cell sorting. Aliquots of CD19+ cells were FACS analysed for B220, IgG1, IgG2a, IgG2b and gB. CD19+ cells corresponding to 1,000 B220+, IgG1+/IgG2a+/IgG2b+, gB+ cells of each genotype were transferred i.v. into 2- to 4-month-old male and female *Rag1*^−*/*−^ mice. Six days later recipient mice were challenged by i.v. injection of 2 μg VLPs and bled regularly.

### ELISA

To measure the gB-specific serum IgG titres, MaxiSorp plates (Nunc) were coated with 0.5 μg ml^−1^ soluble glycoprotein B (gB) of hCMV in 15 mM Na_2_CO_3_, 35 mM NaHCO_3_ (pH 9.6) overnight at 4 °C. Blocking was performed with 5% FCS, 0.05% Tween20 in PBS for at least 2 h at 37 °C or overnight at 4 °C. Plates were incubated with serially diluted serum overnight at 4 °C. Mixed sera of late immunization time points were used as a standard. gB-specific total IgG or IgG1 antibodies were detected by alkaline phosphatase-linked anti-IgG or anti-IgG1 antibodies (Southern Biotechnology), which were incubated on the plates for 2 h at 37 °C.

### ELISPOT

Coating and blocking of plastic plates was done as described for ELISA plates. Cells were serially diluted ranging from 2 × 10^6^ per well to about 7,800 cells per well. Following incubation overnight at 37 °C, cells were discarded and plates were incubated with 1 μg ml^−1^ anti-IgG1-alkaline phosphatase in PBS with 1% gelatin, 1% Tween20 for 1 h at 37 °C. 5-Bromo-4-chloro-3-indolylphosphate was used as substrate for detection.

## Author contributions

N.E. designed the research, performed experiments, supervised the project and wrote the paper, L.M.K. designed and performed experiments and analysed data, W.S. generated and analysed *BTK*-deficient DG75 cells, K.V. performed experiments and analysed data, J.L. generated *GRB2*-deficient DG75 cells, D.R. and T.W. did cell transfer experiments, L.N. provided B-cell-specific *Grb2*-KO mice and analysed data, J.W. analysed the data and wrote the paper.

## Additional information

**How to cite this article:** Engels, N. *et al.* The immunoglobulin tail tyrosine motif upgrades memory-type BCRs by incorporating a Grb2-Btk signalling module. *Nat. Commun.* 5:5456 doi: 10.1038/ncomms6456 (2014).

## Supplementary Material

Supplementary Information Supplementary Figures 1-13.
