# Supplementary material for: The immunoglobulin tail tyrosine motif upgrades memory-type BCRs by incorporating a Grb2-Btk signalling module
Source: Nat Commun. 2014 Nov 21;5:5456. doi: 10.1038/ncomms6456 (PMC4263166; doi:10.1038/ncomms6456)
Supplement: Supplementary Information — Supplementary Figures 1-13. [file ncomms6456-s1.pdf]

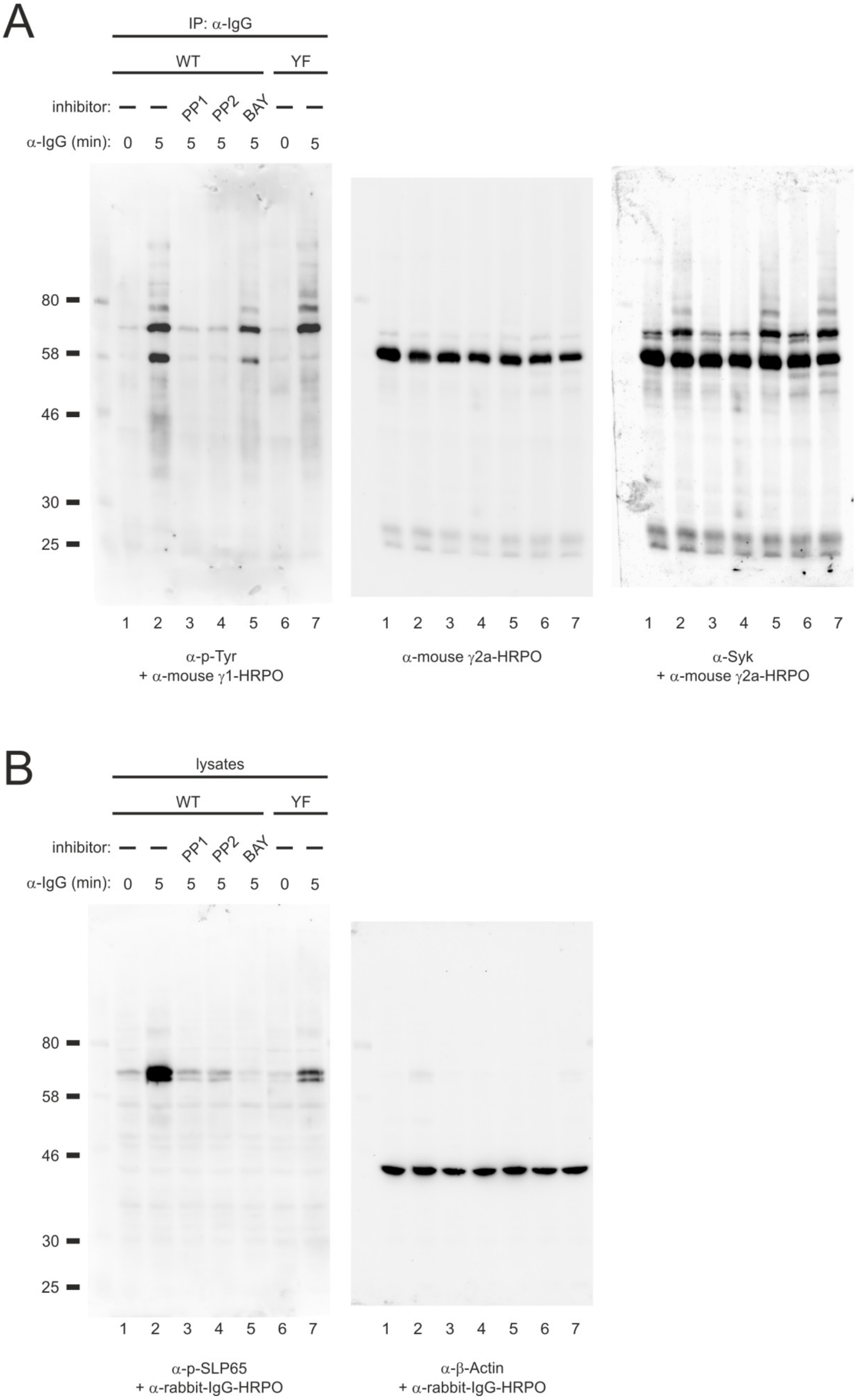

Supplementary Figure 1 Uncropped western blots shown in Figure 2. Nitrocellulose membranes were sequentially developed from left to right.

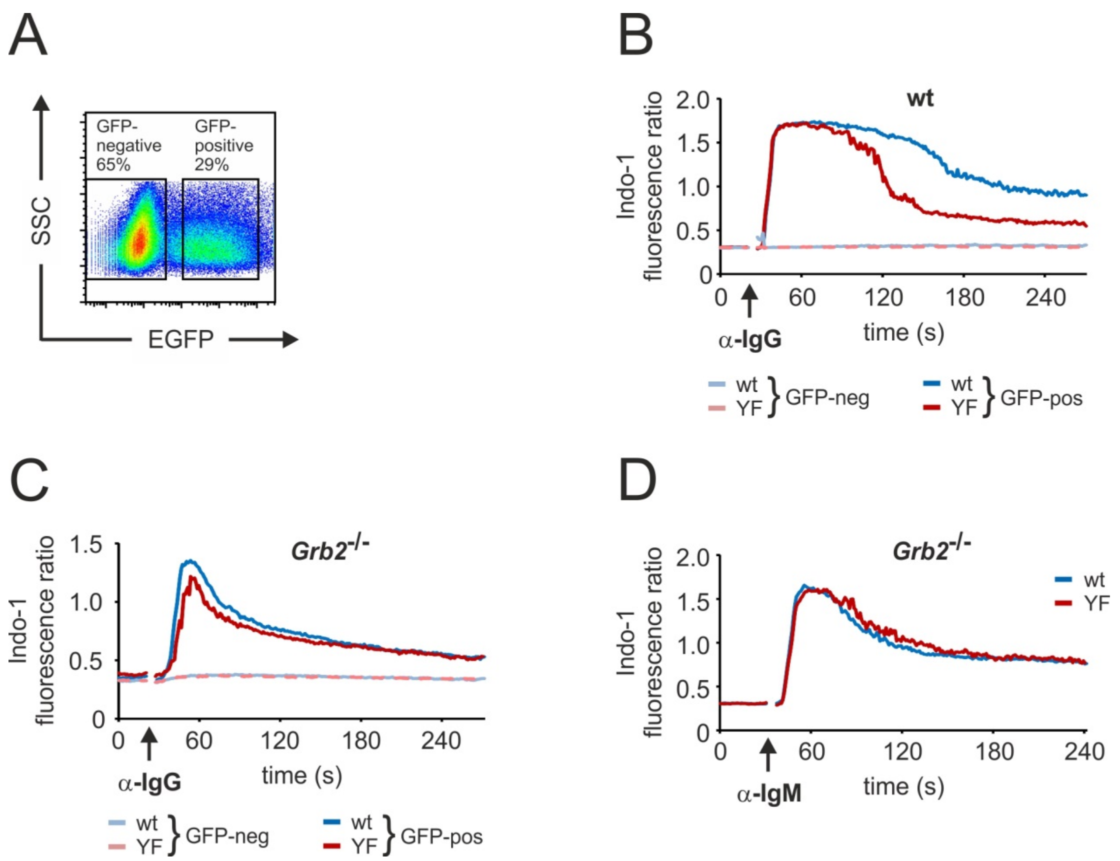

Supplementary Figure 2 Enhanced Ca^{2+} signaling of mIgG-BCRs requires Grb2. Splenic B cells of wild type C57BL/6J mice (**B**, wt) or *Grb2$^{fl/fl}$ mb1$^{cre/+}$* mice (**C** & **D**, *Grb2$^{-/-}$*) were retrovirally transfected to express either wild type (wt, blue lines) or tyrosine to phenylalanine-mutant (YF, red lines) mIgG2a-BCRs along with IRES-driven EGFP (**A**). Cells were stimulated with anti-IgG (**B** & **C**) or anti-IgM (**D**) $F(ab')_2$ fragments (20 mg per ml each) and Ca^{2+} mobilization was recorded in the presence of 1 mM extracellular $CaCl_2$. Data are representative of five independent experiments.

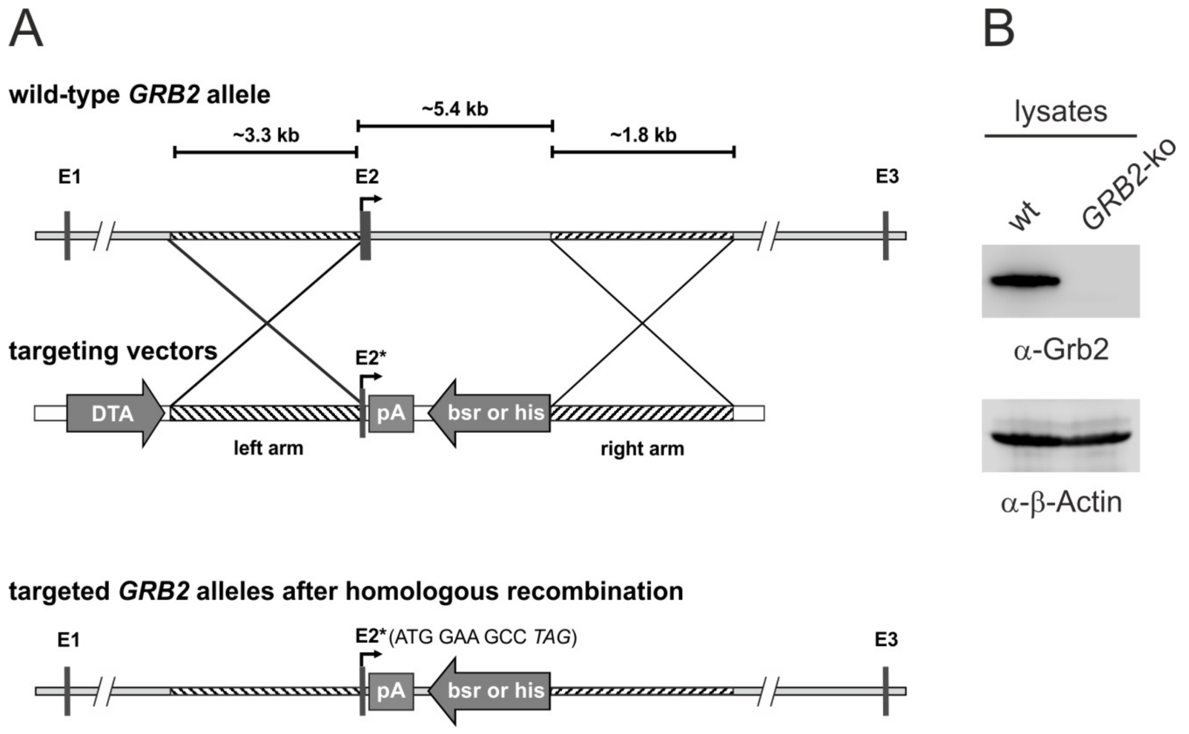

Supplementary Figure 3 Generation of *GRB2*-deficient DG75 cells. (**A**) Two targeting vectors for the two alleles of *GRB2* were generated containing either a blasticidin (bsr) or histidinol (his) resistance cassette, respectively, that replaced parts of exon 2 and in addition introduced a STOP mutation at codon four of the Grb2 open reading frame (E2*), followed by a polyadenylation signal sequence (pA). Furthermore, the targeting vectors contained a diphetria toxin alpha (DTA) cassette from which the toxin is produced if the targeting vectors integrate randomly in the genome. Screening of cells for homologous recombination was done by PCR (data not shown). (**B**) Western blot analysis of Grb2 expression in wild type (wt) and *GRB2*-deficient (*GRB2*-ko) DG75 cells.

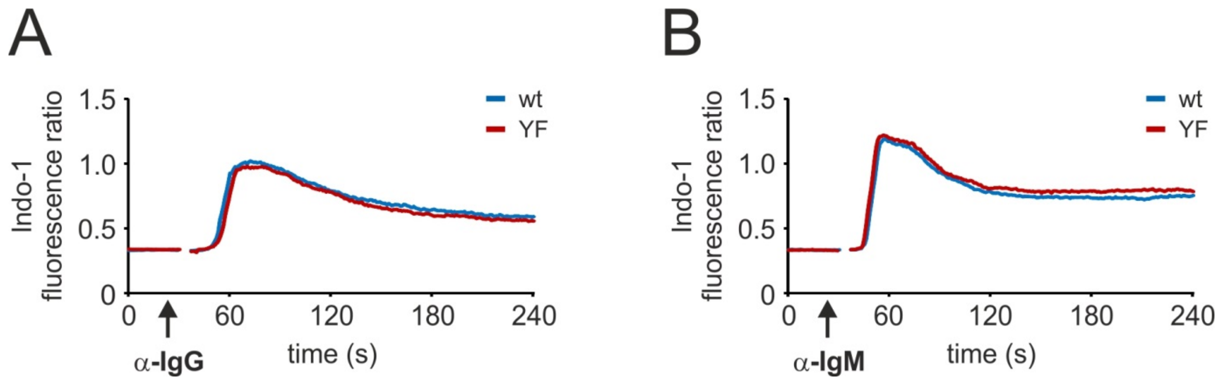

Supplementary Figure 4 Enhanced Ca^{2+} signaling of mIgG-BCRs in human B cells requires Grb2. Wild type (wt, blue lines) and ITT-mutant (YF, red lines) mIgG2a-BCRs were retrovirally expressed in *GRB2*-deficient DG75 B cells. BCR-induced Ca^{2+} mobilization was analyzed upon stimulation with polyclonal $F(ab')_2$ fragments to IgG (**A**) or IgM (20 µg per ml each) to check the integrity of the ITAM-based Ca^{2+} pathway in the transfectants (**B**). Data are representative of two independent experiments.

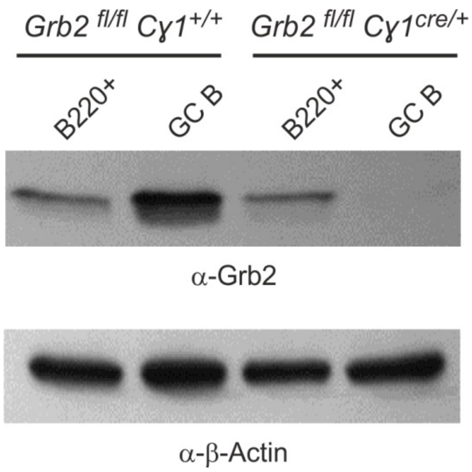

Supplementary Figure 5 Deletion of Grb2 in *Grb2$^{fl/fl}$ Cγ1$^{cre/+}$* germinal center B cells. *Grb2$^{fl/fl}$ Cγ1$^{+/+}$* and *Grb2$^{fl/fl}$ Cγ1$^{cre/+}$* mice were injected with 1 x 10^7 sheep red blood cells each and spleen cells were stained for B220 (clone RA3-6B2), Gl7 (GL-7) and Fas-receptor (Jo2) 10 days later. B220+ GL7- Fas-receptor- (B220+) and B220+ GL7+ Fas-receptor+ germinal center B (GC B) cells were sorted using flow cytometric methods. Lysates were analyzed for expression of Grb2 (clone 3F2) and β-Actin by immunoblotting.

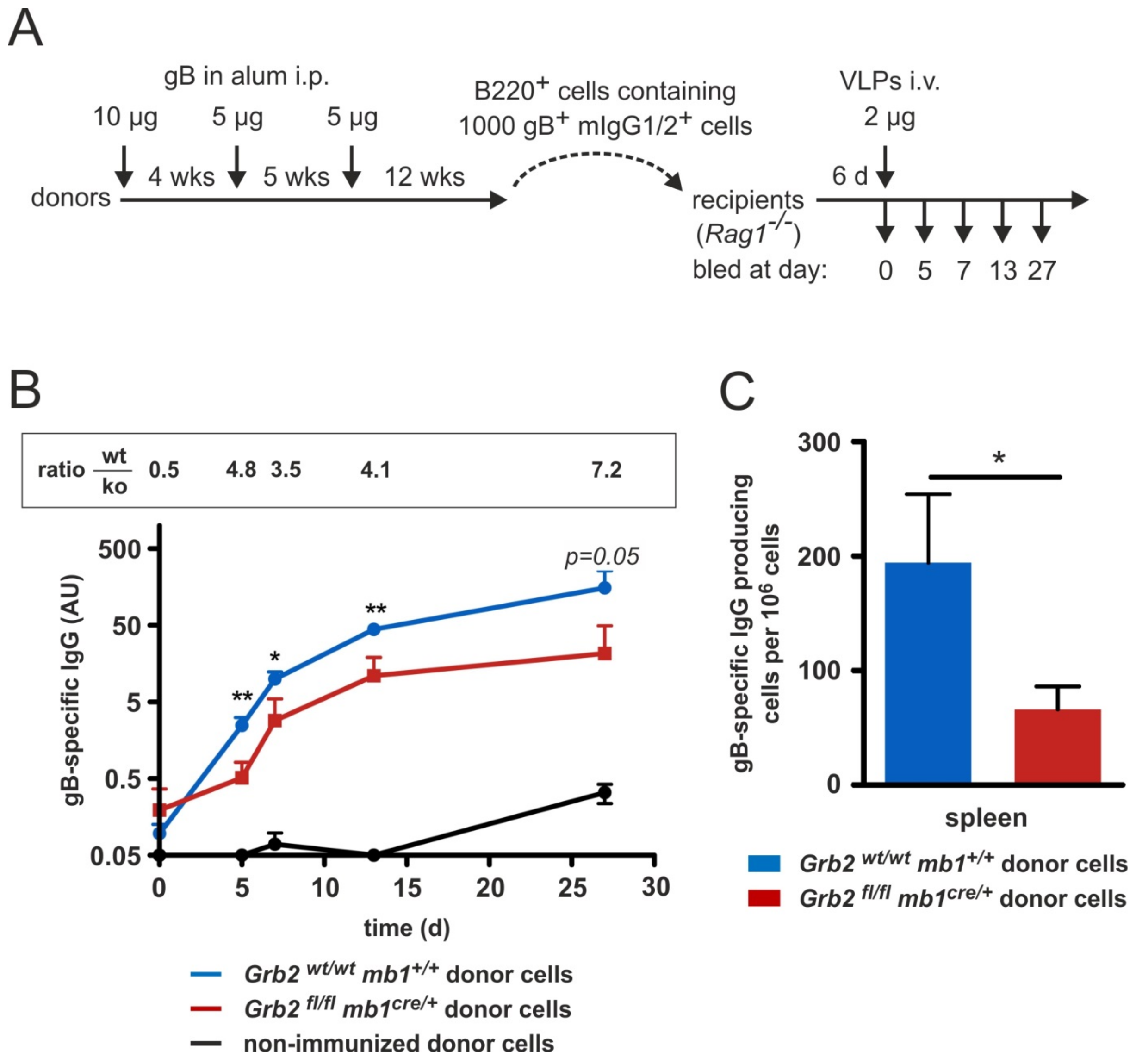

Supplementary Figure 6 T cell-independent reactivation of IgG-switched memory B cells requires Grb2. (**A**) Experimental outline. *Grb2*$^{wt/wt}$*mb1*$^{+/+}$ (n=4) and *Grb2*$^{fl/fl}$*mb1*$^{cre/+}$ mice (n=3) were repeatedly immunized with purified gB in aluminum hydroxide (alum) at the indicated time points. Splenic B cells were purified 83 days after third immunization by complement-mediated T-cell lysis and anti-CD19 magnetic bead separation. CD19 positive cells containing 1000 memory B cells each (identified as B220+, gB+, IgG1+ or IgG2a+ or IgG2c+) were transferred intravenously into *Rag1*$^{-/-}$ recipient mice and challenged six days later by an intravenous injection of 2 µg virus like particles (VLPs) of human CMV in PBS. (**B**) gB-specific IgG titers were measured by ELISA. The ratio of IgG titers produced by wild type cells vs. *Grb2*-ko cells in given. (**C**) ELISPOT for gB-specific IgG-secreting cells was performed at day 27 after VLP challenge. Error bars represent mean + SD; students T-test was used. *p<0.05 **p<0.01

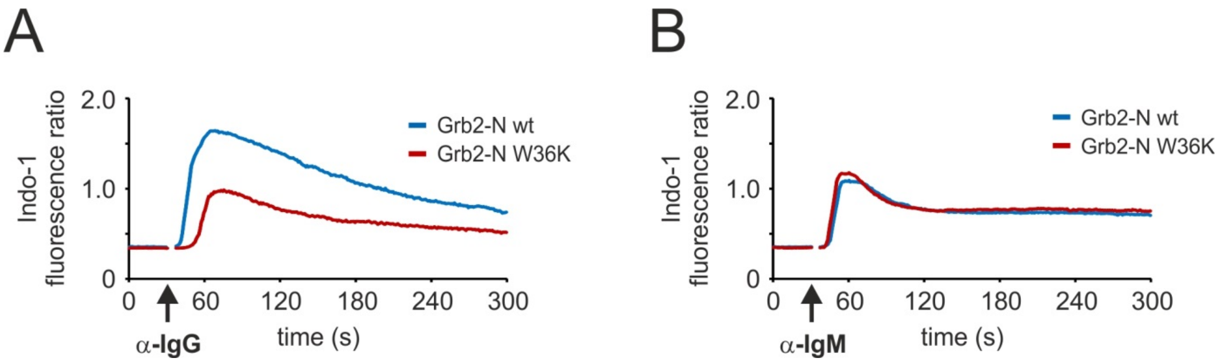

Supplementary Figure 7 Signal amplification of the ɣ2am-YF-Grb2-N-SH3 chimera does not require endogenous Grb2. Chimeric ɣ2am-YF-Grb2-N-SH3 constructs (wild type, wt, blue lines or with inactivated SH3 domain, W36K, red lines) were retrovirally expressed in *GRB2*-deficient DG75 B cells. BCR-induced Ca^{2+} mobilization was analyzed upon stimulation with polyclonal $F(ab')_2$ fragments to IgG (**A**) or IgM to check the integrity of the ITAM-based Ca^{2+} pathway in the transfectants (**B**). Data are representative of two independent experiments.

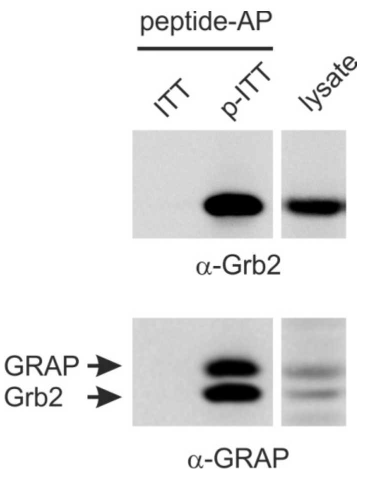

Supplementary Figure 8 The ITT binds Grb2 and GRAP. Biotinylated peptides encompassing the ITT sequence (BIOT-NH2-KQTISPDYRNMIGQ-COOH) were used in unphosphorylated (ITT) or tyrosine-phosphorylated (p-ITT) form for affinity purifications of binding partners from lysates of DG75 B cells using a streptavidin matrix. Purified proteins were separated by SDS-PAGE and blotted to a nitrocellulose membrane. The blot was sequentially developed with antibodies to Grb2 (upper panel) and GRAP (lower panel). The anti-GRAP antibody crossreacts with the closely related Grb2 protein. Data are representative of three independent experiments.

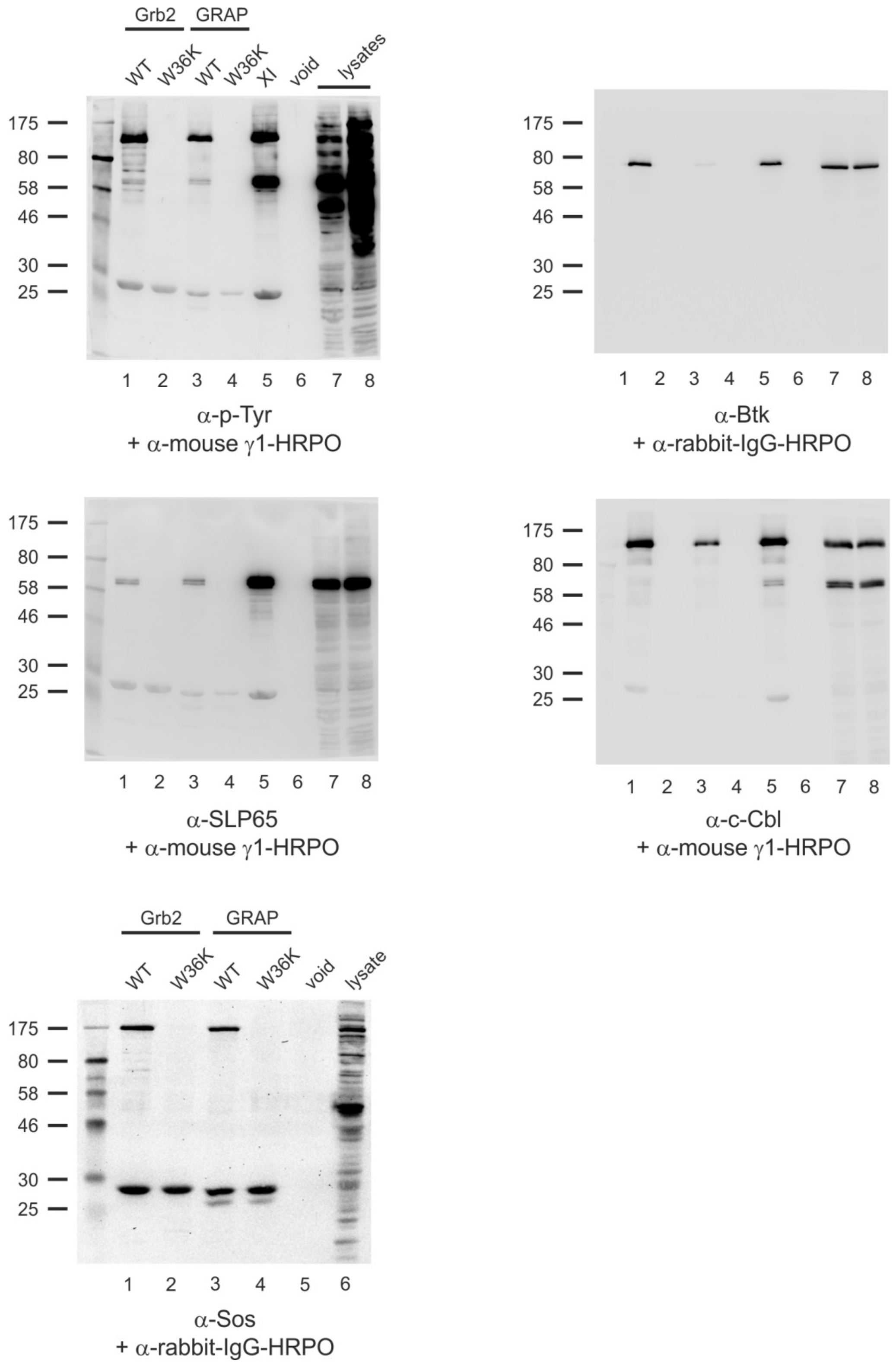

Supplementary Figure 9 Uncropped western blots shown in Figure 6. "XI" denotes a chimeric Grb2/GRAP SH3 domain (see Supplementary Figure 12). This lane is not shown in Figure 6 as it was not relevant there.

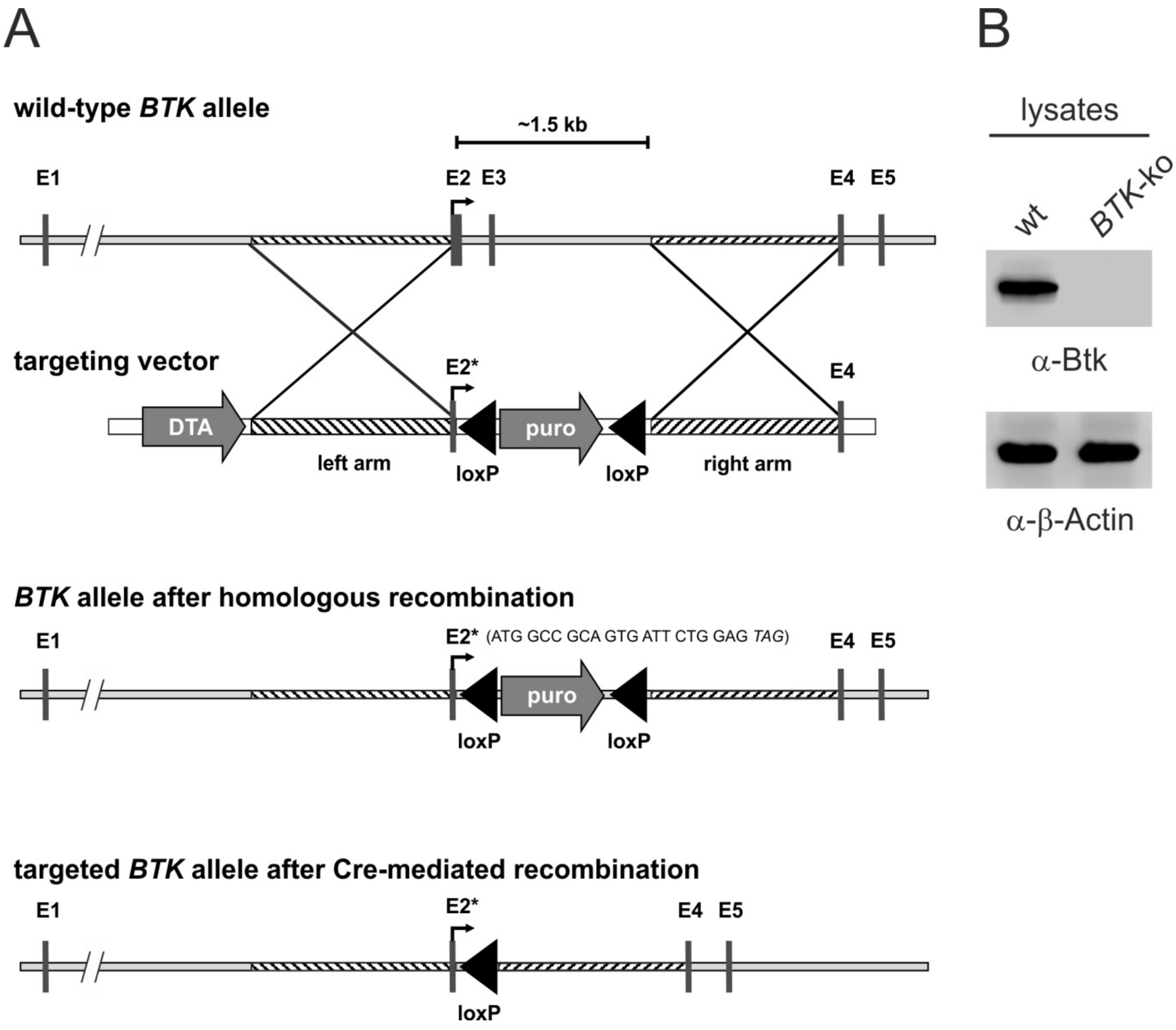

Supplementary Figure 10 Generation of *BTK*-deficient DG75 cells. DG75 Burkitt lymphoma cells were obtained from a boy with a primary abdominal lymphoma. Since in humans the *BTK* gene is located on the X chromosome, DG75 cells contain only a single copy of that gene. (**A**) Targeting strategy of human *BTK* gene. The targeting vector contained a loxP site-flanked puromycin (puro) resistance cassette that replaced parts of exon 2 and the entire exon 3 of the *BTK* gene. In addition a STOP mutation was introduced at the eighth codon of the open reading frame in exon 2 (E2*). After successful homologous recombination, cells were transiently transfected with an expression vector for the Cre recombinase to allow for excision of the puromycin resistance cassette. Eventually, the modified *BTK* locus contained a truncated, mutant exon 2 and a remnant loxP site. (**B**) Western blot analysis of Btk expression in wild type (wt) and *BTK*-deficient (*BTK*-ko) DG75 cells.

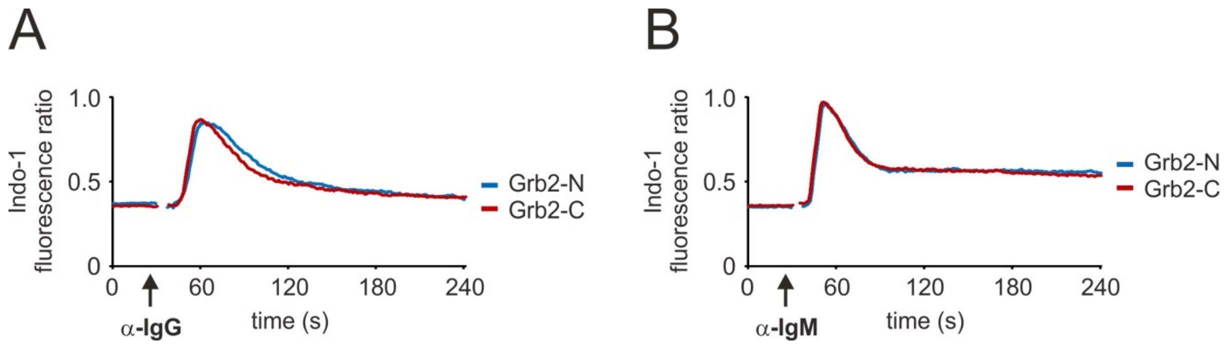

Supplementary Figure 11 Signal amplification of the ɣ2am-YF-Grb2-N-SH3 chimera depends on the expression of Btk. Chimeric ɣ2am-YF-Grb2-SH3 constructs (N-terminal SH3 domain, Grb2-N, blue lines or C-terminal SH3 domain, Grb2-C, red lines) were retrovirally expressed in *BTK*-deficient DG75 B cells. BCR-induced Ca^{2+} mobilization was analyzed upon stimulation with polyclonal $F(ab')_2$ fragments to IgG (**A**) or IgM to check the integrity of the ITAM-based Ca^{2+} pathway in the transfectants (**B**). Data are representative of three independent experiments.

A

```
hGrb2     MEAIAKYDFKATADDELSFKRGDILKVLNEECDQNWYKAELNGKDGFIPKNYIEMKPH 58
hGRAP     MESVALYSFQATESDELAFNKGDTLKILNMEDDQNWYKAELRGVEGFIPKNYIRVKPH 58
          **::* *.*:** .***:*::** **:** * *********.* :********.:***

XI        MEAIAKYDFKATADDELSFKRGDILKILNMEDDQNWYKAELRGVEGFIPKNYIRVKPH 58

XII       MEAIAKYDFKATESDELAFNKGDTLKILNMEDDQNWYKAELRGVEGFIPKNYIRVKPH 58

XIII      MEAIAKYSFQATESDELAFNKGDTLKILNMEDDQNWYKAELRGVEGFIPKNYIRVKPH 58

XIV       MEAIALYSFQATESDELAFNKGDTLKILNMEDDQNWYKAELRGVEGFIPKNYIRVKPH 58

XV        MEAVALYSFQATESDELAFNKGDTLKILNMEDDQNWYKAELRGVEGFIPKNYIRVKPH 58
```

B

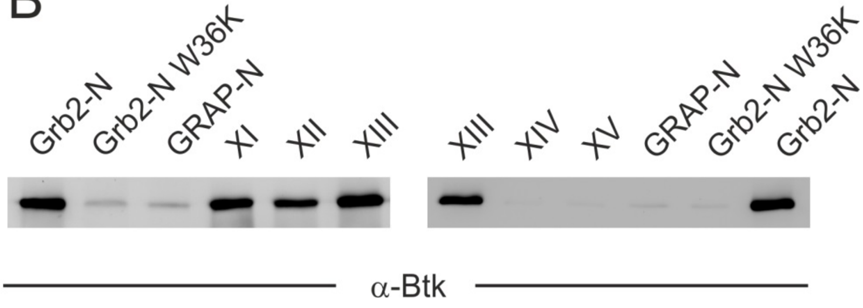

Supplementary Figure 12 Identification of Btk-binding amino acid residues in the N-terminal SH3 domain of Grb2. (**A**) Amino acid sequences of the N-terminal SH3 domains of human Grb2, GRAP and various chimeras thereof (designated as XI-XV). (**B**) Affinity purifications with GST-coupled SH3 domains shown in (A) from lysates of human B cells were analyzed with anti-Btk antibodies. Data are representative of three independent experiments.

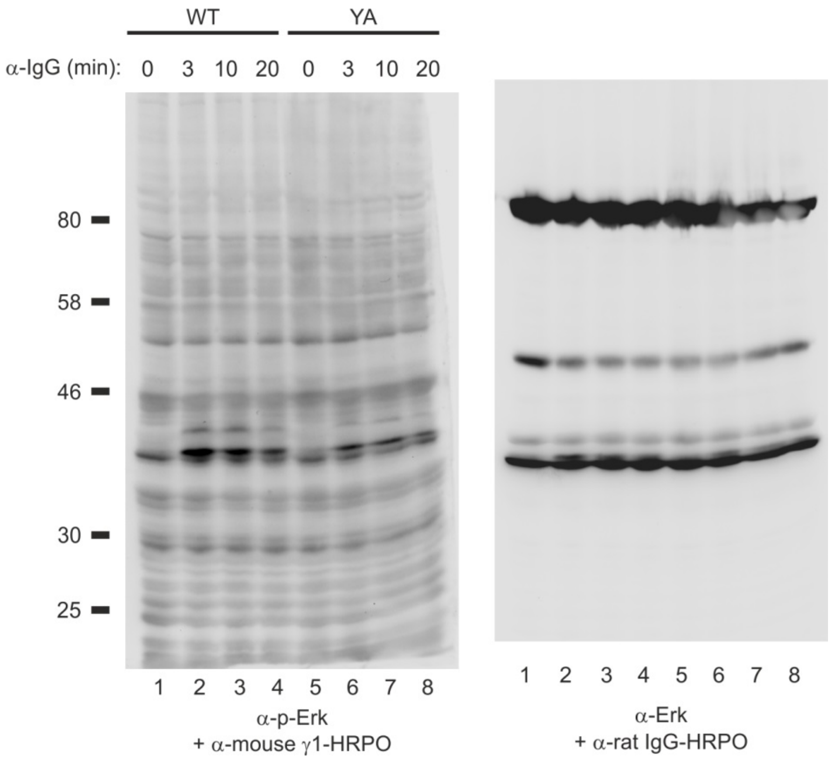

Supplementary Figure 13 Uncropped western blots shown in Figure 8B. The nitrocellulose membrane was sequentially developed with antibodies against phospho-Erk1/2 (α-p-Erk) and pan-Erk (α-Erk).
